# Author Correction: Genomic profile of advanced breast cancer in circulating tumor DNA

**DOI:** 10.1038/s41467-021-24791-5

**Published:** 2021-07-16

**Authors:** Belinda Kingston, Rosalind J. Cutts, Hannah Bye, Matthew Beaney, Giselle Walsh-Crestani, Sarah Hrebien, Claire Swift, Lucy S. Kilburn, Sarah Kernaghan, Laura Moretti, Katie Wilkinson, Andrew M. Wardley, Iain R. Macpherson, Richard D. Baird, Rebecca Roylance, Jorge S. Reis-Filho, Michael Hubank, Iris Faull, Kimberly C. Banks, Richard B. Lanman, Isaac Garcia-Murillas, Judith M. Bliss, Alistair Ring, Nicholas C. Turner

**Affiliations:** 1 The Breast Cancer Now Toby Robins Research Centre, The Institute of Cancer Research, London, UK; 2 Centre for Molecular Pathology, Royal Marsden Hospital, London, UK; 3 ICR-CTSU, The Institute of Cancer Research, London, UK; 4 NIHR Manchester Clinical Research Facility at The Christie, Manchester Academic Health Science Centre & Division of Cancer Sciences, School of Medical Sciences, Faculty of Biology Medicine & Health, University of Manchester, Manchester, UK; 5 The Beatson West of Scotland Cancer Centre, Glasgow, UK; 6 Cancer Research UK Cambridge Centre, Cambridge, UK; 7 University College London Hospitals NHS Foundation Trust, London, UK; 8 Memorial Sloan Kettering Cancer Centre, New York, NY USA; 9 Guardant Health, Inc., Redwood City, CA USA; 10 Ralph Lauren Centre for Breast Cancer Research, Royal Marsden Hospital, London, UK

**Keywords:** Translational research, Breast cancer, Cancer genetics

Correction to: *Nature Communications* 10.1038/s41467-021-22605-2, published online 23 April 2021.

The original version of this Article contained an error in Fig. 4. In Fig. 4b the asterisks denoting significance were incorrectly shown as red instead of black. This error has been corrected in the PDF and HTML versions of the Article.

