# Supplementary material for: Genomic profile of advanced breast cancer in circulating tumour DNA
Source: Nat Commun. 2021 Apr 23;12:2423. doi: 10.1038/s41467-021-22605-2 (PMC8065112; doi:10.1038/s41467-021-22605-2)
Supplement: Supplementary file 1 — Supplementary Information [file 41467_2021_22605_MOESM1_ESM.pdf]

Genomic Profile of Advanced Breast Cancer in Circulating Tumor DNA

Supplemental Figures and Tables

Supplementary Figures 1 - 16

Supplementary Tables 1 - 4

Supplementary Fig. 1. plasmaMATCH trial design

a

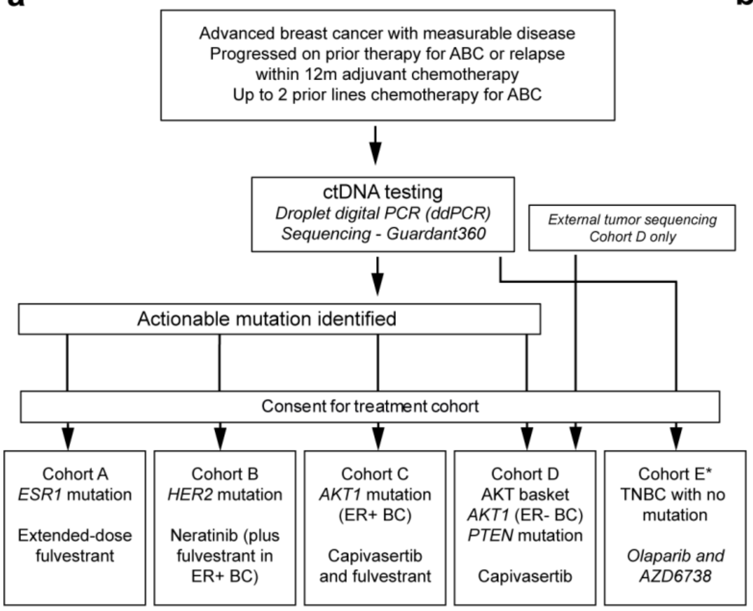

b

DNA testing	ctDNA		
	Droplet digital PCR (ddPCR)		Targeted Sequencing Guardant360
Testing	Prospectively tested		Prospectively and retrospectively tested
Location	Centralised at Centre for Molecular Pathology, Royal Marsden		Guardant Health
n	1025		800 (364/436)
Gene Inclusion	*PIK3CA* multiplex:	E542K	*AKT1 ALK APC AR ARAF ARID1A ATM BRAF BRCA1 BRCA2 CCND1 CCND2 CCNE1 CDH1 CDK4 CDK6 CDKN2A DDR2 EGFR ERBB2 ESR1 EZH2 FBXW7 FGFR1 FGFR2 FGFR3 GATA3 GNA11 GNAQ GNAS HNF1A HRAS IDH1 IDH2 JAK2 JAK3 KIT KRAS MAP2K1 MAP2K2 MAPK1 (ERK2) MAPK3 (ERK1) MET MLH1 MPL NF1 NFE2L2 NOTCH1 NPM1 NRAS NTRK1 NTRK3 PDGFRA PIK3CA PTEN PTPN11 RAF1 RB1 RET RHEB RHOA RIT1 ROS1 SMAD4 SMO SRY STK11 TERT TP53 TSC1 VHL*
		E545K	
		H1047R	
		H1047L	
	ESR1 multiplexes:	E380Q	
		L536R	
		Y537C	
		D538G	
		S463P	
		Y537N	
		Y537S	
	AKT1 singleplex:	E17K	
	HER2 singleplex:	S310F	
		S310Y	
		L755S	
		V777L	
		GSP insertion *(P780_Y781insGSP)*	
		YVMA insertion *(A775_G776insYVMA)*	

a) PlasmaMATCH trial scheme: Advanced breast cancer patients with disease progression after prior therapy, and following consent, were offered ctDNA testing by droplet digital PCR (ddPCR) for the presence of actionable mutations in *ESR1, HER2, PIK3CA* and *AKT1* or targeted sequencing. Patients were enrolled into one of the five interventional arms based on the mutations detected in plasma, which is reported separately[1], with cohort E *ongoing.

b) Circulating tumor DNA (ctDNA) testing for patients in plasmaMATCH. Droplet digital PCR (ddPCR) tested for hotspot mutations in *ESR1*, *HER2*, *PIK3CA* and *AKT1* while targeted sequencing employed a duplex sequencing 73/74 gene panel (Guardant360)[2]. Of 800 patients who underwent targeted sequencing, 364 did so prospectively as part of the trial, and 436 retrospectively from banked baseline plasma (“Methods”).

Supplementary Fig. 2: Degree of elevated gene copy number in circulating tumor DNA

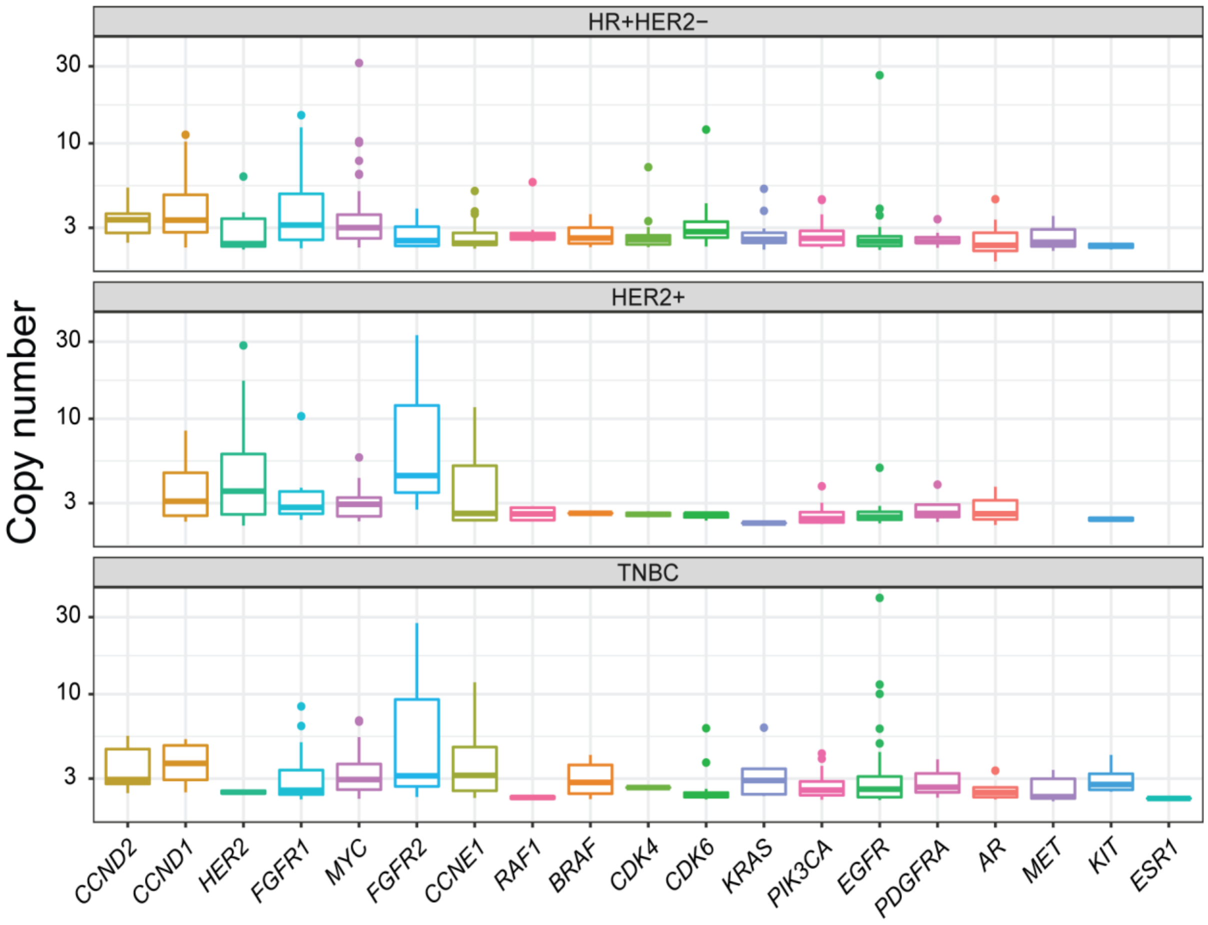

Copy number (CN) in plasma for indicated genes in patients with elevated plasma copy number, by breast cancer subtype, to identify genes likely amplified at high copy number (HR+HER2- N=515, HER2+ N=72, TNBC N=138). Data are presented as a boxplot, where the middle line is the median, the lower and upper hinges represent the 25th and 75th centiles respectively and the whiskers extend from the hinge to the smallest and largest value, respectively, no further than 1.5 x IQR (interquartile range) from the lower or upper hinge. Data outside of these ranges are plotted individually.

Supplementary Fig. 3: Sensitivity and specificity of Guardant360 targeted sequencing

All mutation calls		ddPCR Positive	ddPCR Negative
Targeted Sequencing	Positive	492	77
	Negative	49	9841

	%
Sensitivity	90.9
Specificity	99.2
ppv	86.5
npv	99.5

ddPCR AF<1%		ddPCR Positive	ddPCR Negative
Targeted Sequencing	Positive	161	77
	Negative	38	9841

	%
Sensitivity	80.9
Specificity	99.2
ppv	67.6
npv	99.6

Sensitivity and specificity of targeted panel sequencing (Guardant360) compared to droplet digital PCR (ddPCR) in N=682 patients who underwent testing for hotspot mutations within *PIK3CA, ESR1, HER2* and *AKT1* (Supplementary Fig. 1b) using both technologies. The upper table represents all mutations at all allele frequencies (AF). The lower table includes mutations with ddPCR AF<1%. The sensitivity of targeted sequencing in identifying mutations is 90.9%, and remains high at 80.9% for mutations at low AF.

Supplementary Fig. 4: Co-occurrence and mutual exclusivity of mutations in HR+HER2- breast cancer

HR+HER2-

	ESR1	*PIK3CA*	*TP53*	*GATA3*	*ARID1A*	*PTEN*	*HER2*	*NF1*	*KRAS*	*AKT1*	*RB1*
RB1											
AKT1											
KRAS		0.022									
NF1						0.023					
HER2				0.009							
PTEN			0.023								
ARID1A											
GATA3		0.028									
TP53	0.002										
PIK3CA											
ESR1											

Mutually Exclusive: q value < 0.05; p value < 0.05

Co-occurent: q value < 0.05; p value < 0.05

Association analysis for most frequent mutated genes in HR+HER2- breast cancer (N=515 patients) with two-sided Fisher's exact test p values. Green genes showing mutual exclusivity, and purple co-occurrence with dark colours indicating significance following false discovery correction.

Supplementary Fig. 5: Correlation of *ESR1* and *MAPK* pathway mutation allele frequency from the same patient

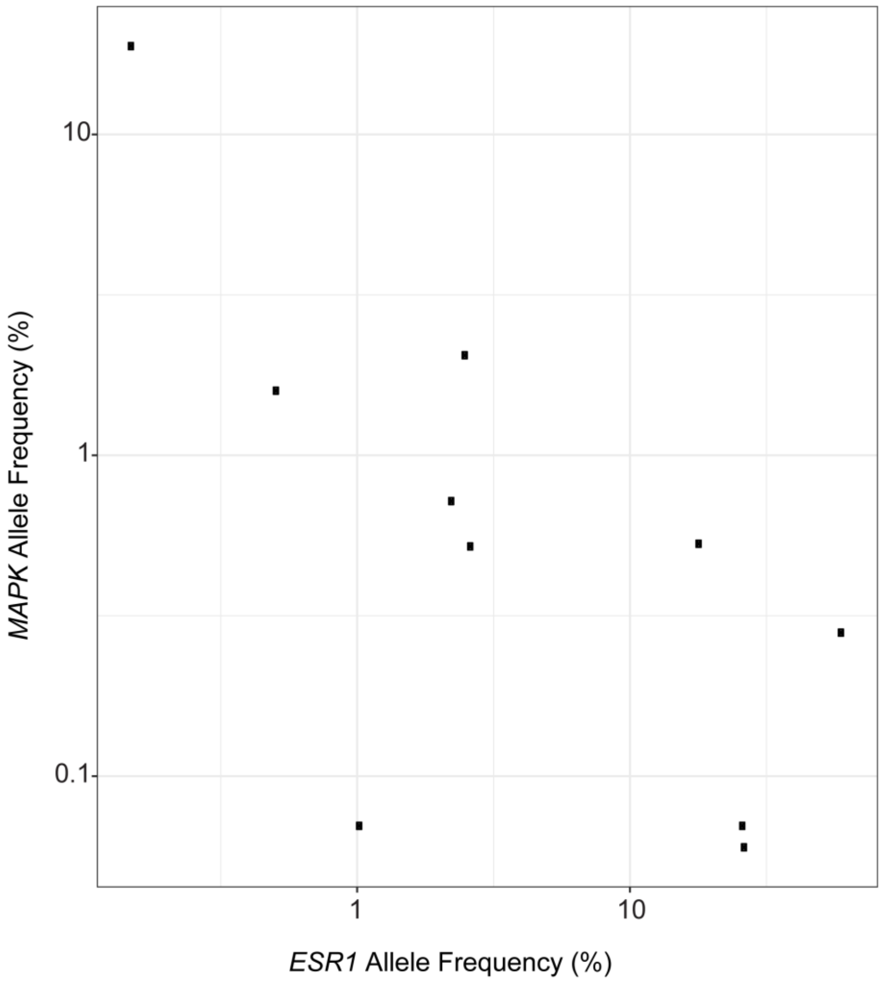

Correlation of allele frequency of *ESR1* and *MAPK* pathway mutations from the same patient, in patients with single *ESR1* and *MAPK* mutations (N=10). Spearman correlation coefficient -0.264, p=0.017 (two-sided). Includes all patients with HR+HER2- disease and single mutations in both.

Supplementary Fig. 6: Mutational profile of plasmaMATCH compared to TCGA

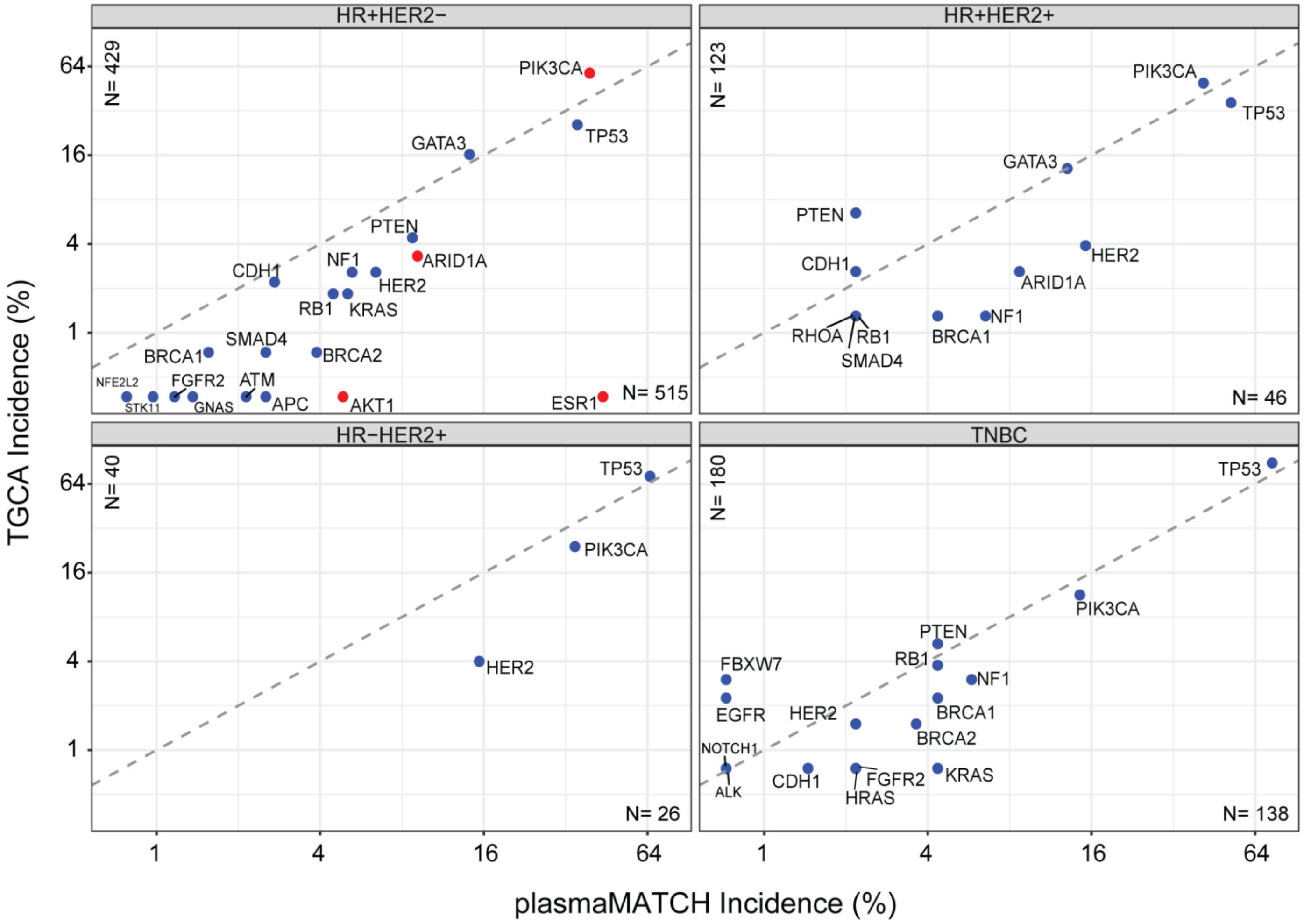

Mutational profile of ctDNA in plasmaMATCH compared to published large primary breast cancer tissue sequencing dataset (TCGA)[3]. Red dots indicate significant change in frequency after false discovery adjusted two-sided Fishers exact test (HR+HER2-: *ESR1* q<0.0001, *PIK3CA* q<0.0001, *AKT1* q=0.006, *ARID1A* q=0.04). Included are genes with an incidence 1.5% in both data sets. N indicates the number of patients included in each group.

Supplementary Fig. 7: Validation of rare and hotspot mutations in *HER2* by droplet digital PCR

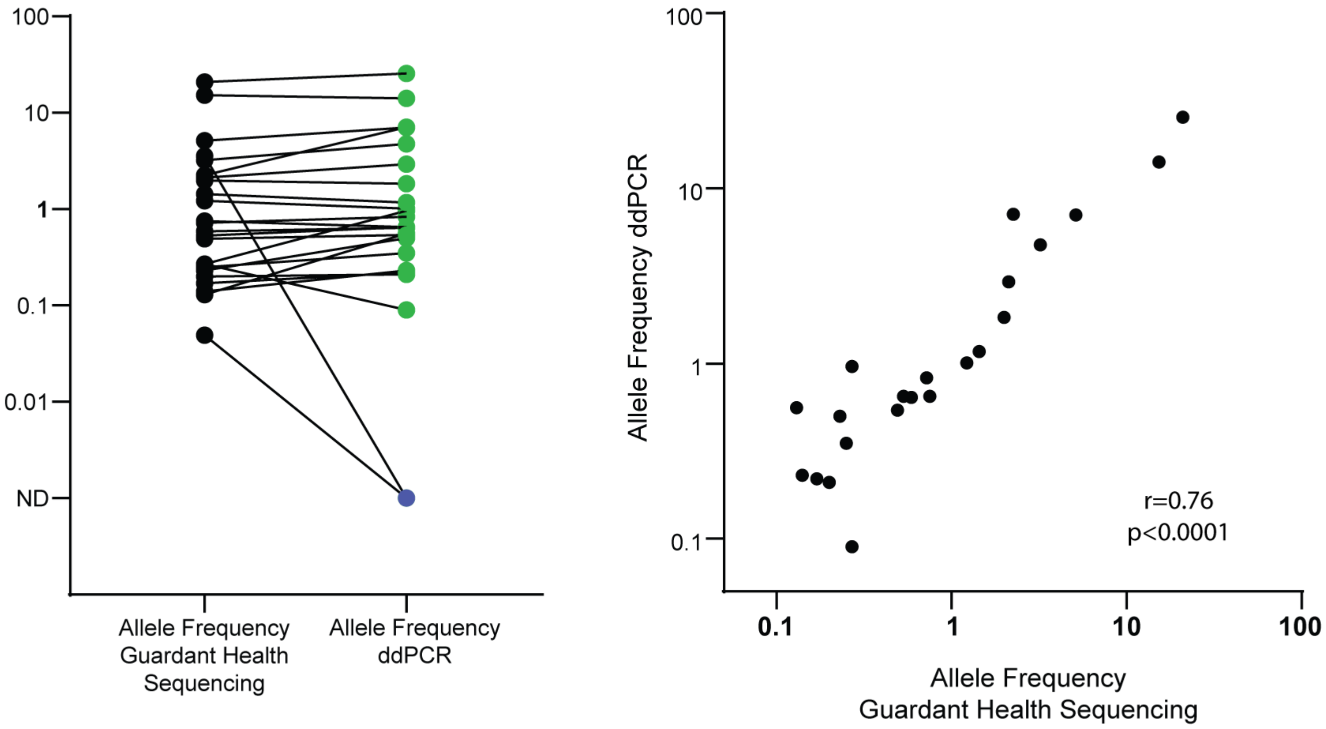

Association between allele frequency in ctDNA sequencing and validation analysis with plasma DNA droplet digital PCR (ddPCR), N=24 *HER2* mutation assays. 22/24 (91.7%) mutations were validated by ddPCR. Spearman correlation coefficient 0.76, P<0.0001 (two-sided). ND, not detected.

Supplementary Fig. 8: Prior therapy and mutations prevalence in HR+HER2- advanced breast cancer

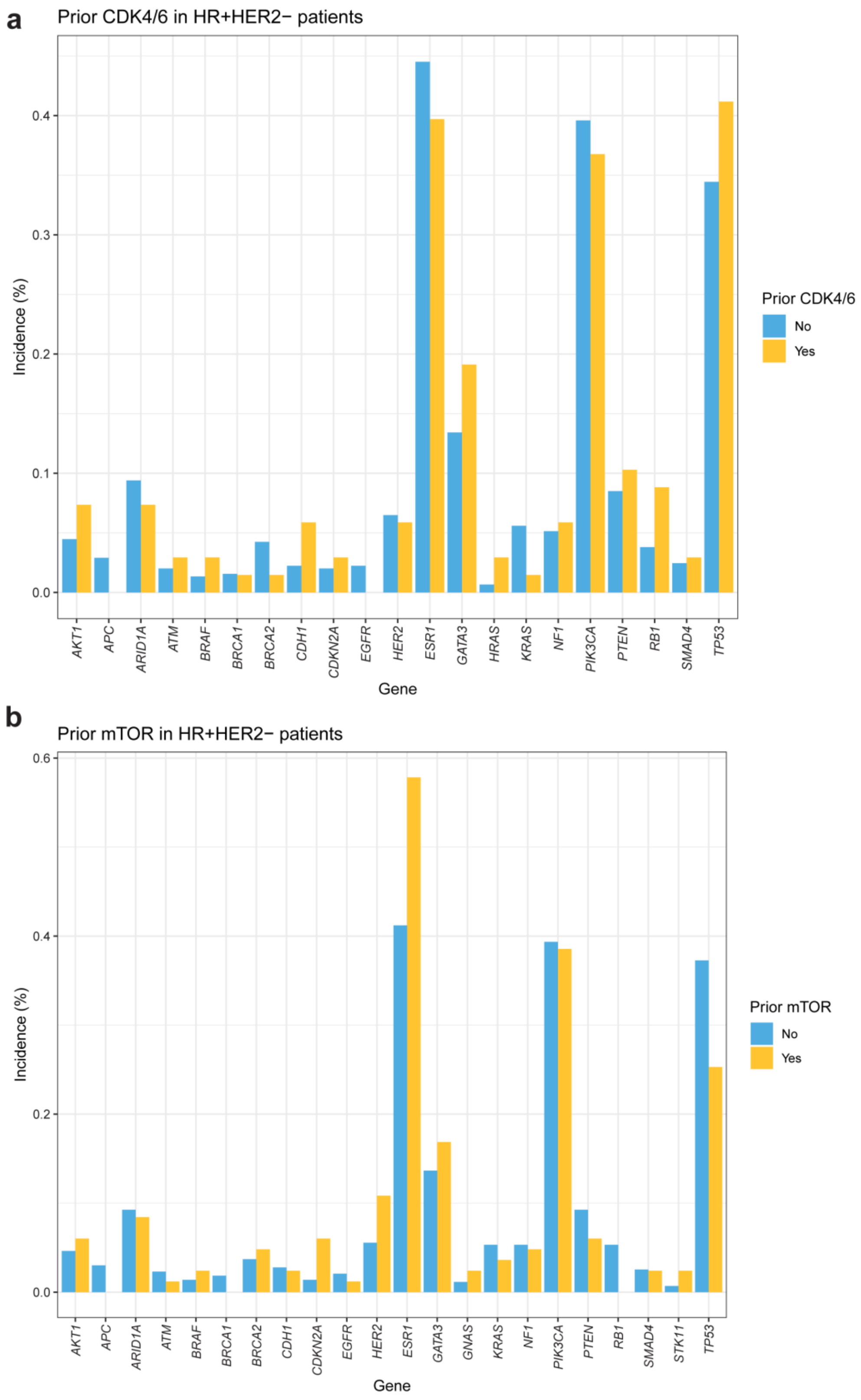

a) Mutation incidence in HR+HER2- breast cancer with (N=68 patients) and without (N=447 patients) prior exposure CDK4/6 inhibitor. Genes with an incidence of 1.5% or more in either group are shown.

b) Mutation incidence in HR+HER2- breast cancer with (N=83 patients) and without (N=432 patients) prior exposure mTOR inhibitor everolimus.

Supplementary Fig. 9: Cis-trans analysis of *ESR1* mutations

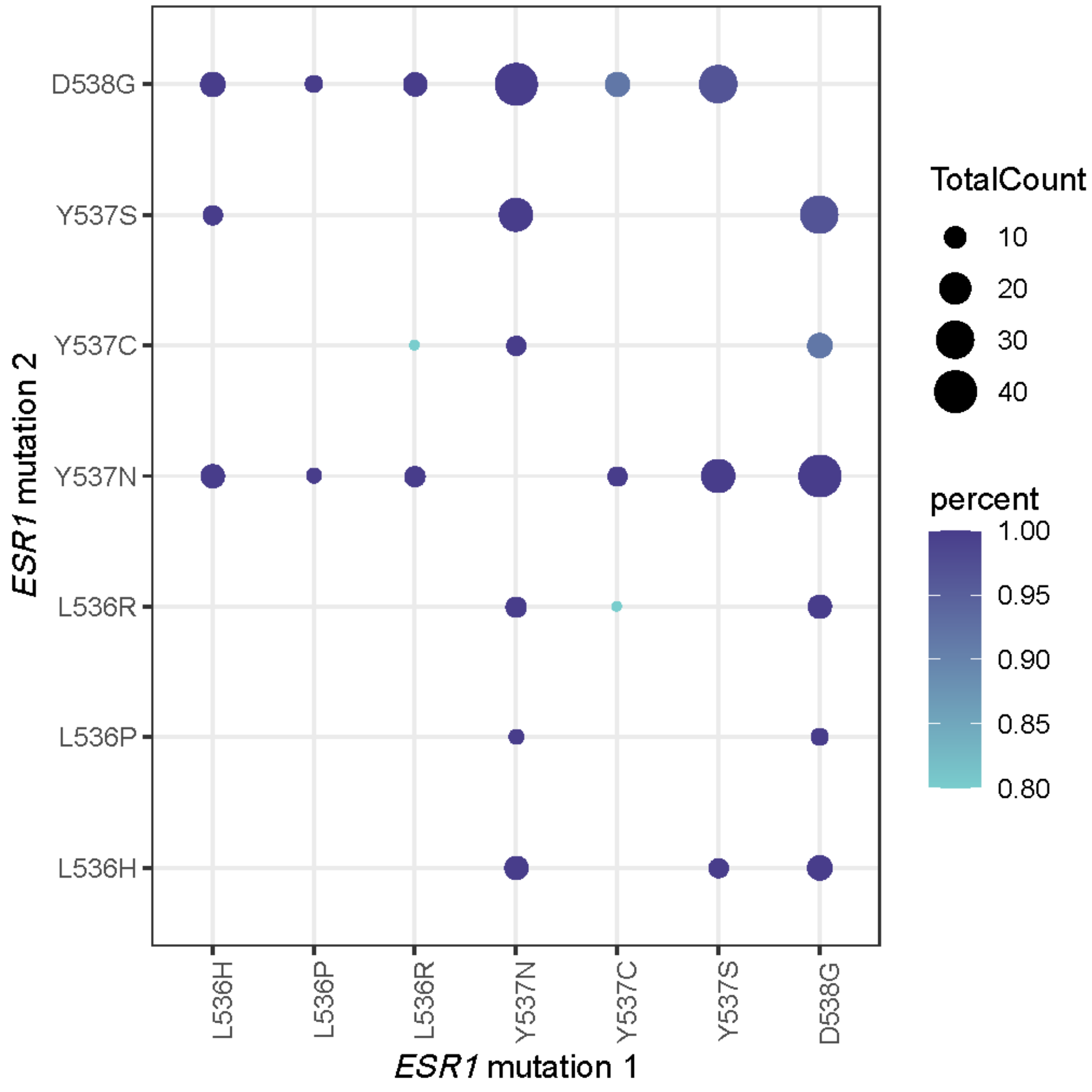

In patients with assessable multiple *ESR1* mutations (N=290), the fraction of mutation pairs that occur in trans.

Supplementary Fig. 10: Clonality of mutations with time from diagnosis of early breast cancer

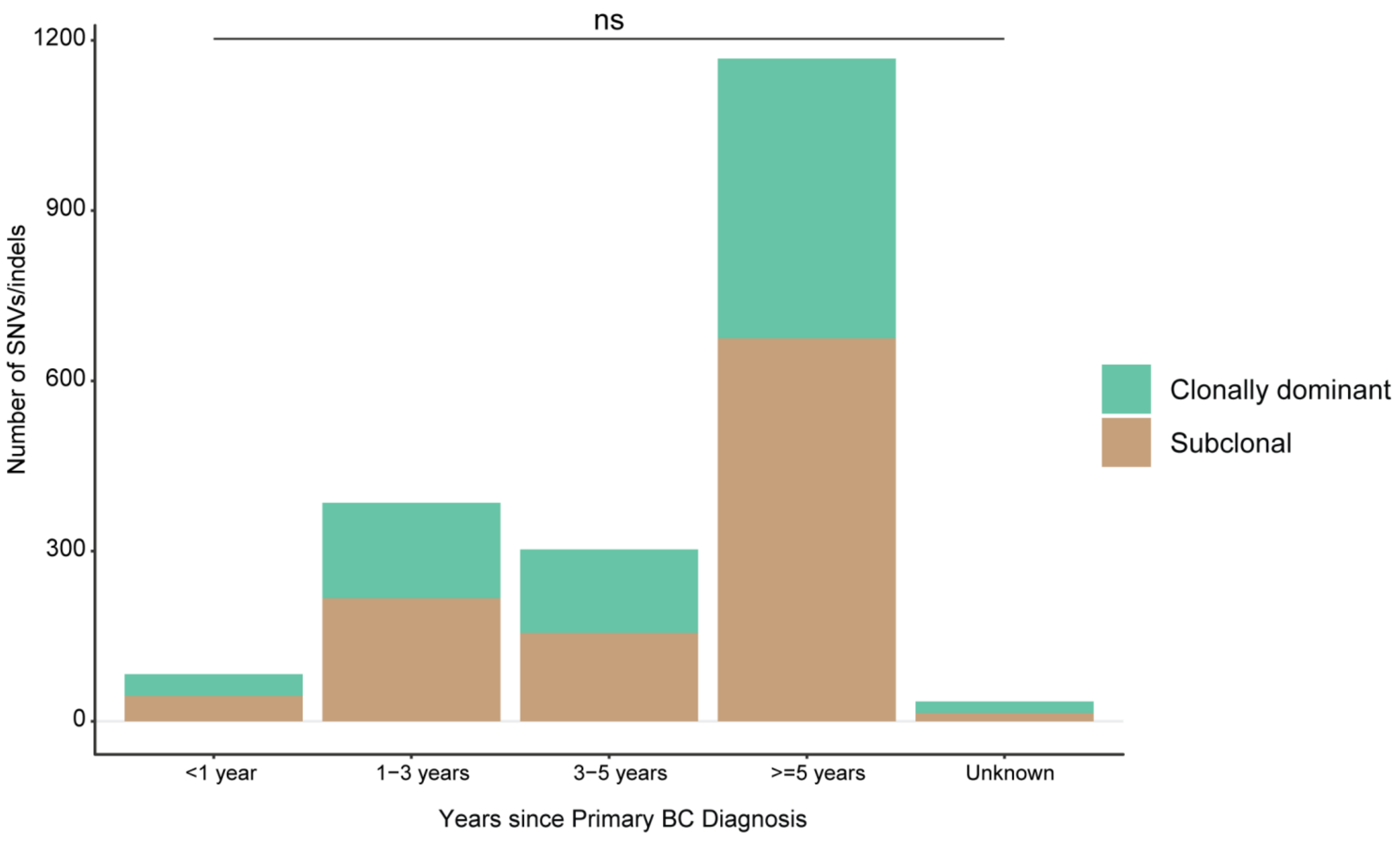

The proportion of clonally dominant to subclonal mutations (N=1974 SNVs/indels with assessable clonality) does not significantly alter with time from diagnosis of early breast cancer (p=0.3, Chi-squared test). ns, not significant

Supplementary Fig. 11. Mutational signature analysis in HER2 positive breast cancer

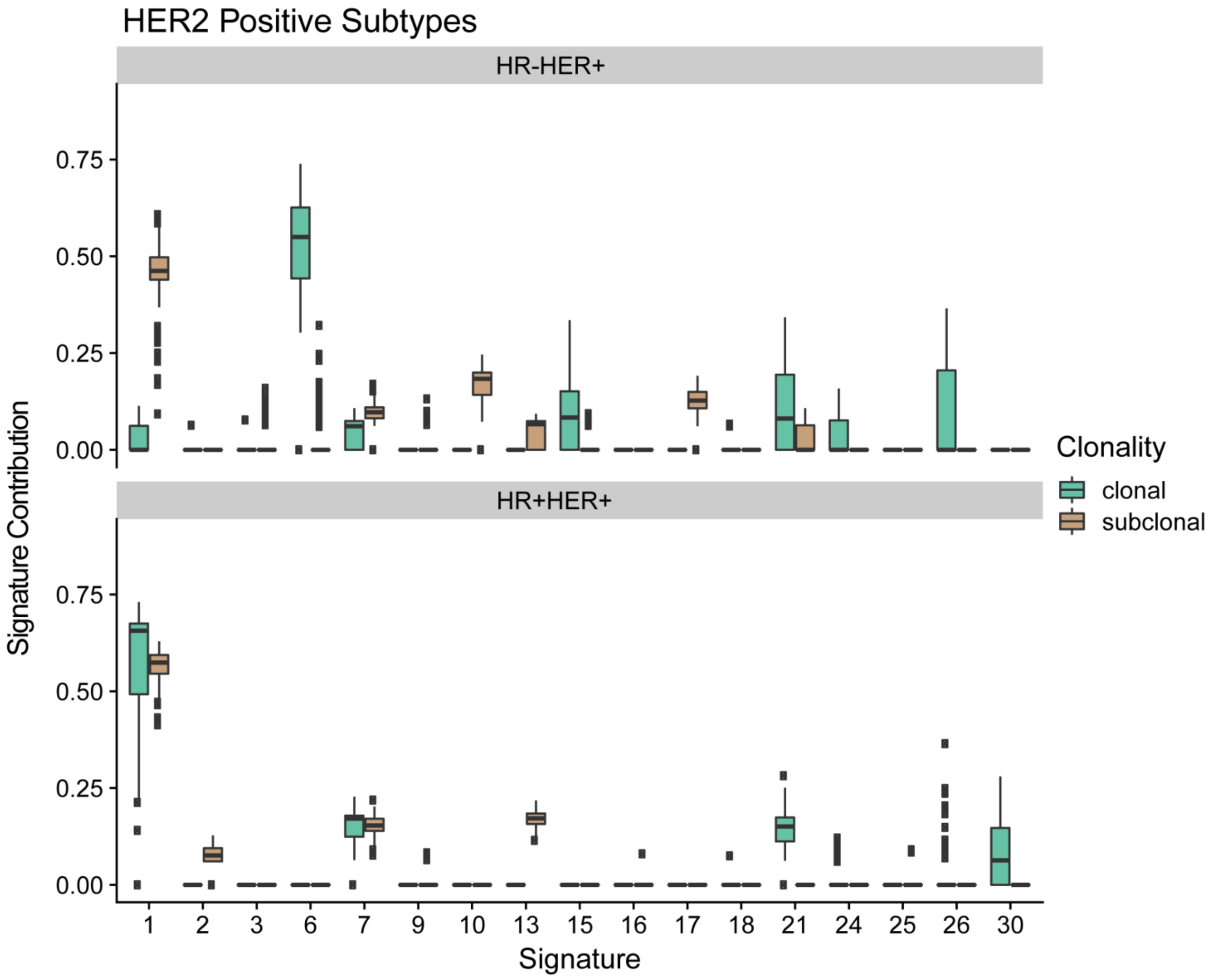

Bootstrap mutational signature analysis on aggregated mutations from all HR-HER2+ (*top*, N=29 clonal mutations, N=29 subclonal mutations) and HR+HER2+ (*bottom,* N=41 clonal mutations, N=97 subclonal mutations) and breast cancers, for clonally dominant and subclonal mutations. Data are presented as a boxplot, where the middle line is the median, the lower and upper hinges represent the 25th and 75th centiles respectively and the whiskers extend from the hinge to the smallest and largest value, respectively, no further than 1.5 x IQR (interquartile range) from the lower or upper hinge. Data outside of these ranges are plotted individually. Although analysis is underpowered, similar patterns are observed compared to TNBC and HR+HER- disease, respectively.

Supplementary Fig. 12: SigMA analysis of mutational signatures in clonally dominant and subclonal mutations in HR+HER2- and TNBC disease

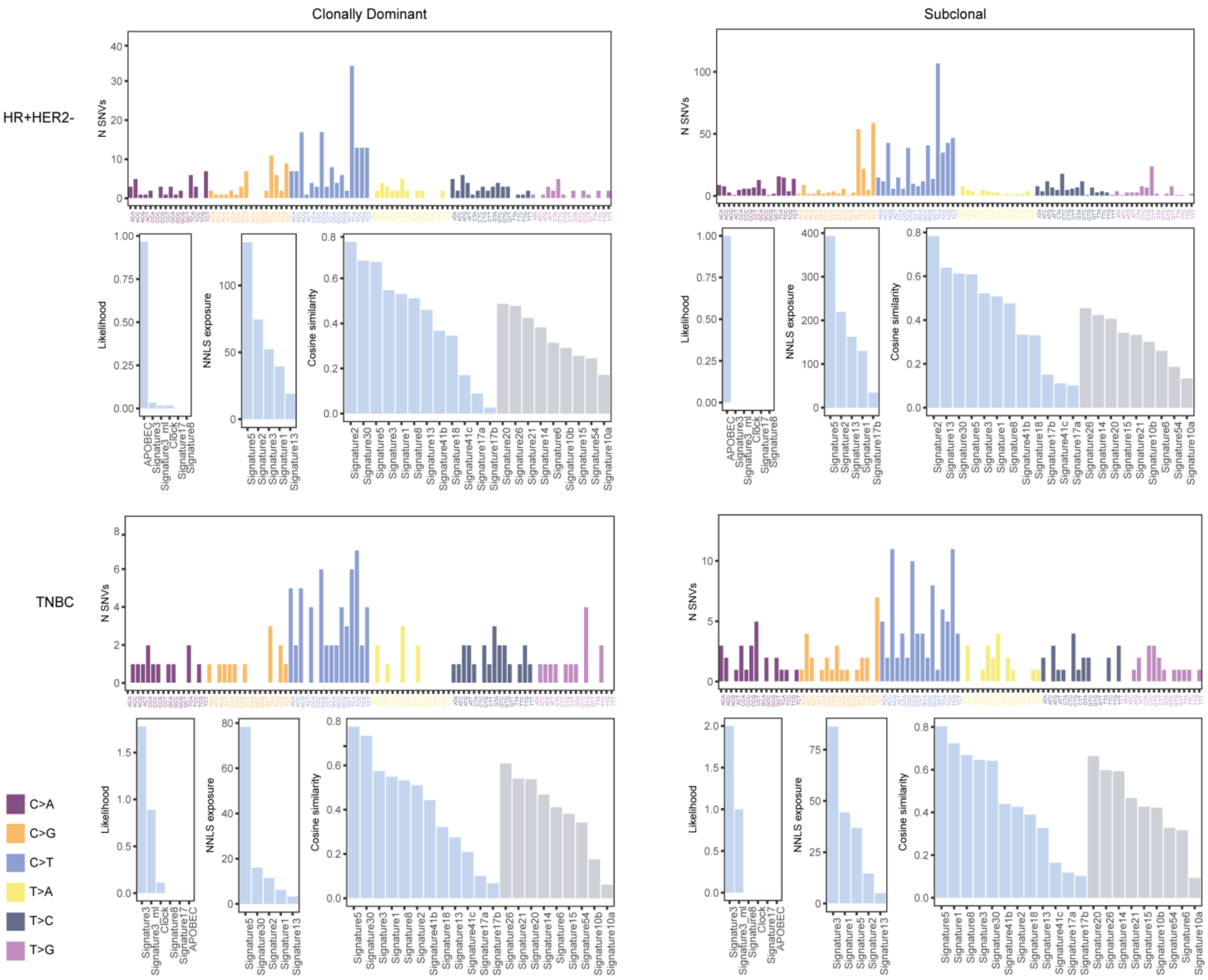

Signature analysis using SigMA[4] for clonally dominant (HR+HER2- N=328; TNBC N=121) and subclonal mutations (HR+HER2- N=968; TNBC N=190) in HR+HER- and TNBC breast cancer. NNLS (non-negative least squares) exposure and Cosine similarity are two orthogonal methods which identify mutational signatures within sequencing data, whilst Likelihood describes the mutational signatures ascertained as present by the SigMA software. SigMA identifies APOBEC as strongly present in all HR+HER2- disease, whilst NNLS exposure and Cosine similarity both identify

signature 13 more strongly in subclonal disease than in clonally dominant disease. In TNBC, age-related signature 1 is more strongly identified by NNLS exposure and Cosine similarity in subclonal disease than clonally dominant disease.

Supplementary Fig. 13: Validation of novel subclonal mutations in *PIK3CA* by droplet digital PCR

a

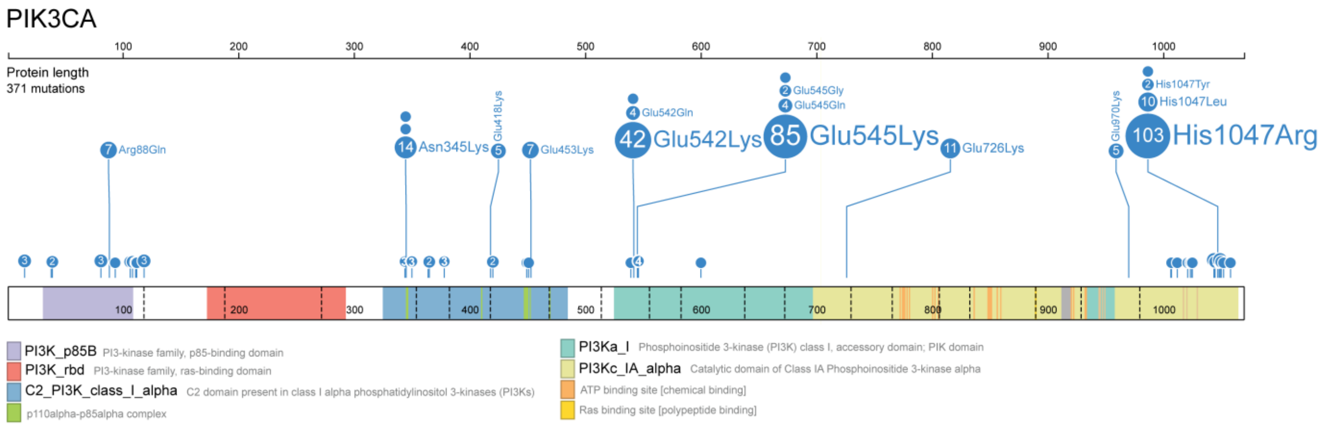

b

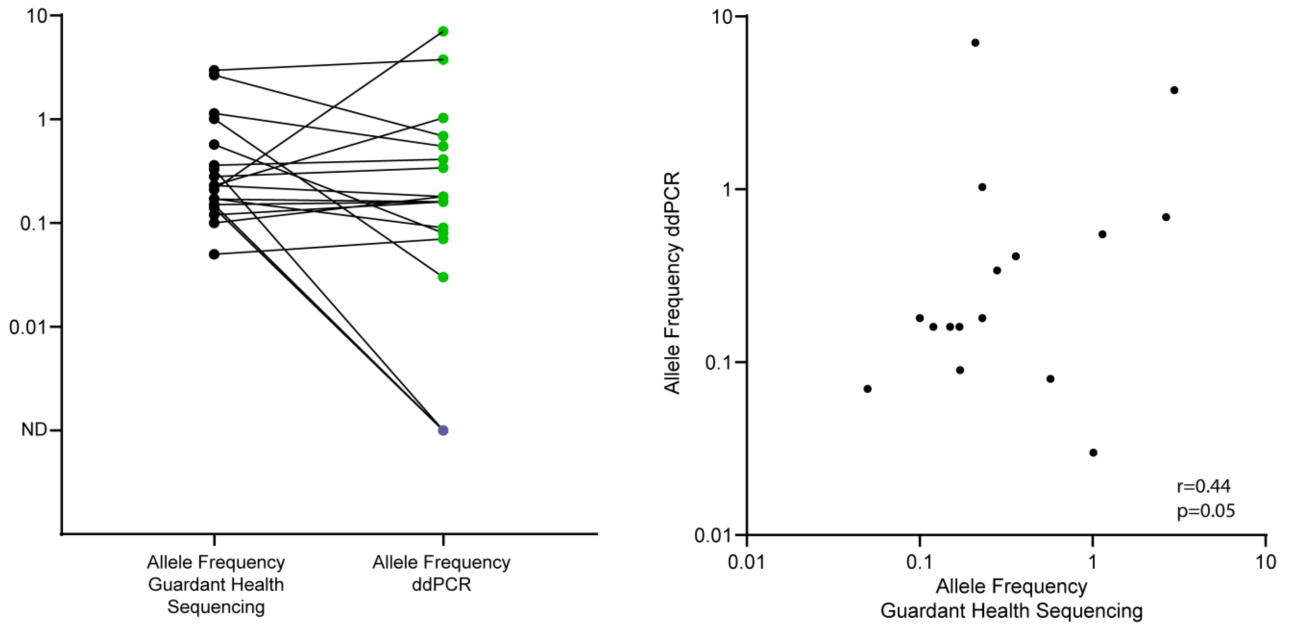

a) PIK3CA mutations at both common and novel hotspots. Illustration from https://proteinpaint.stjude.org[5].

b) Association between allele frequency in ctDNA sequencing and validation analysis with plasma DNA droplet digital PCR (ddPCR), n=20 *PIK3CA* mutation assays. 16/20 (80.0%) of mutations were validated by ddPCR. Spearman correlation coefficient 0.44, P=0.05 (two-sided). ND, not detected.

Supplementary Fig. 14: Analysis of *PIK3CA* mutation multiplicity

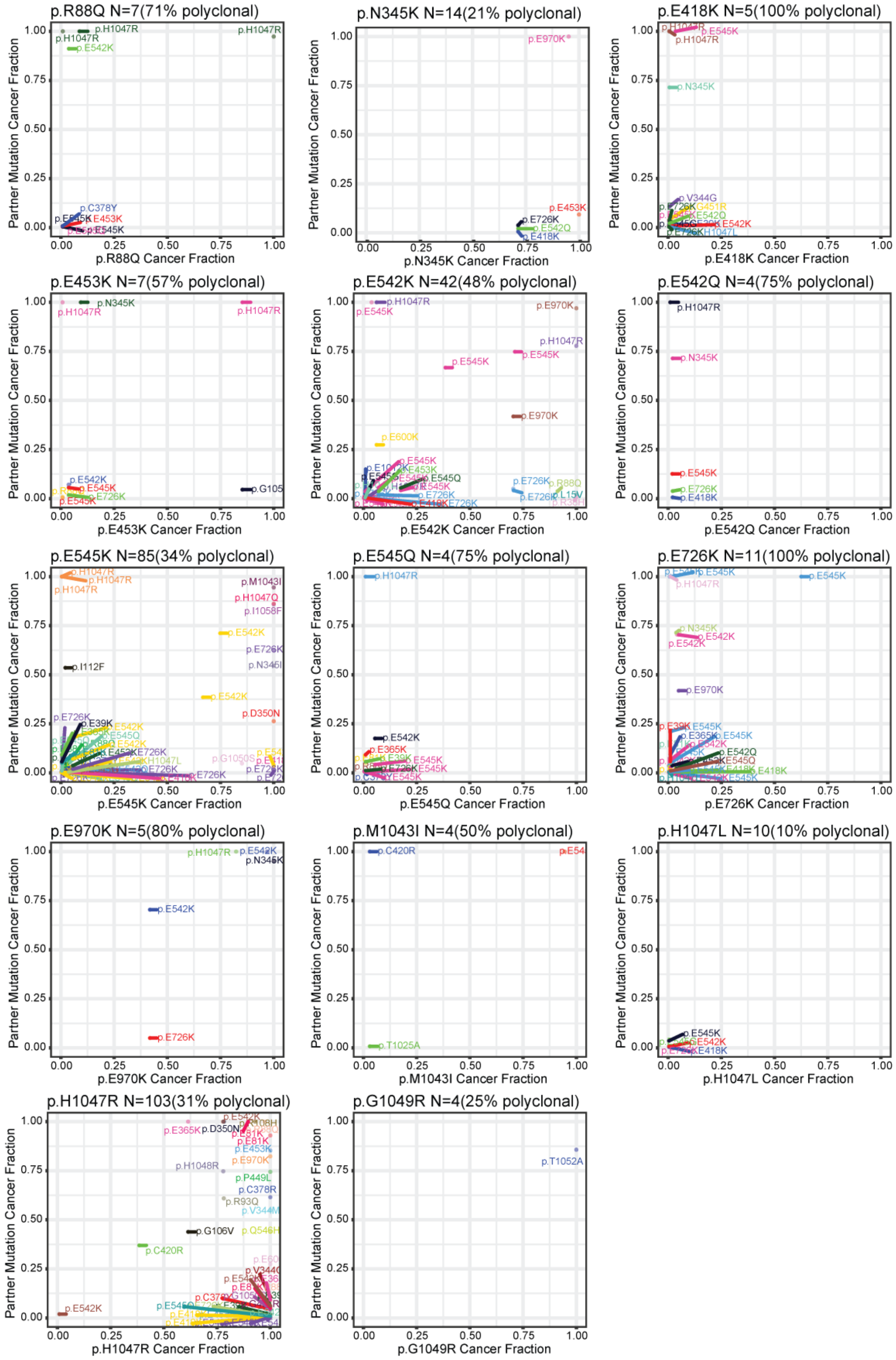

For each individual *PIK3CA* mutation, the proportion of patients with that mutation with multiple *PIK3CA* mutations, and plot of individual *PIK3CA* mutation cancer fraction versus indicated partner *PIK3CA* mutation.

Supplementary Fig. 15: Classes of *PIK3CA* mutation in advanced breast cancer

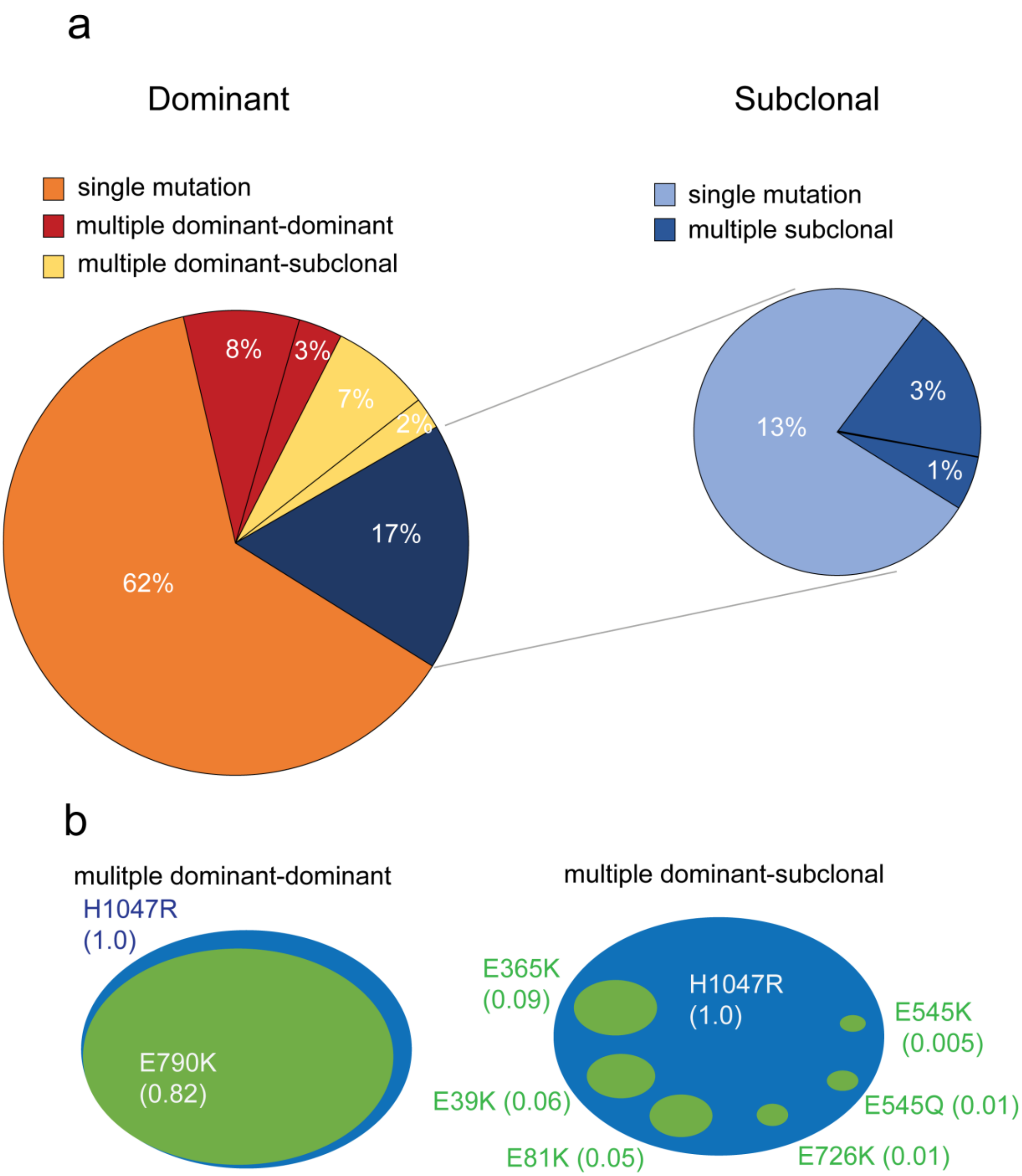

a) *PIK3CA* mutation classes in ctDNA from 293 patients with *PIK3CA* mutations detected.

Left 83% patients (242) have clonally dominant *PIK3CA* mutations, with 62% (183) having a single dominant mutation, 11% having at least two dominant mutations (multiple dominant-dominant: 8% [23] with 2 mutations, 3% [9] with >2 mutations) and 9% having a single dominant mutation with second subclonal mutations (multiple dominant-subclonal: 7% [20] have 2 subclonal mutations and 2% [7] >2 subclonal mutations). Future research will be required to investigate whether response rates to PI3 kinase inhibition vary by class.

Right 17% (51) have subclonal *PIK3CA* mutations, with 13% (38) having a single subclonal mutation and 4% have multiple subclonal mutations (multiple subclonal: 9 with 2 mutations, and 4 with multiple)

b) Examples cancer fractions of two patients; *left* multiple dominant-dominant class in a patient with two clonally dominant mutations in ctDNA; *right* multiple dominant-subclonal class in a patient with a single clonally dominant mutation and multiple subclonal mutations. Number indicates cancer fraction for indicated mutation.

Supplementary Fig. 16: Association between allele frequency and copy number in ctDNA sequencing

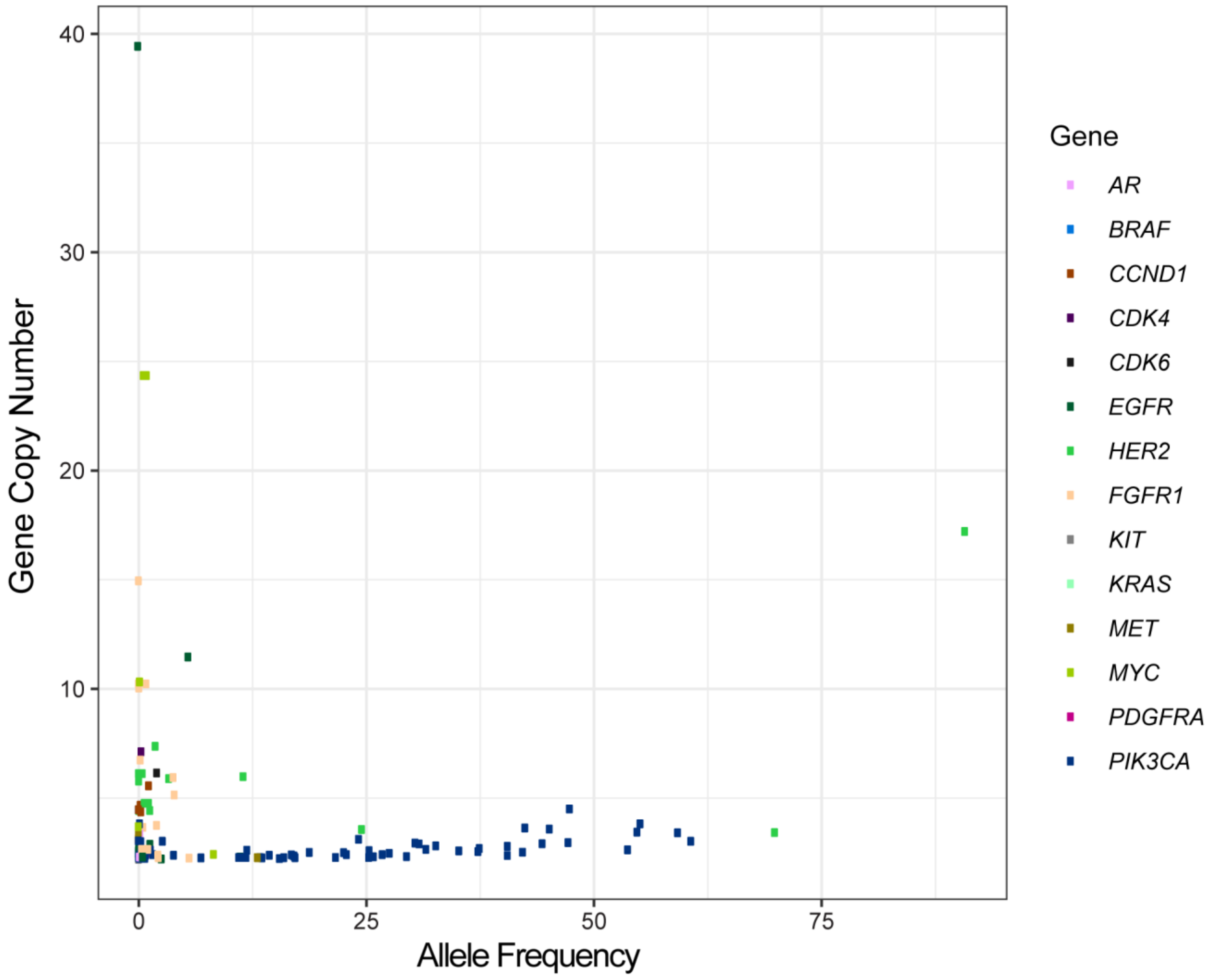

No association between mutation allele frequency and copy number, for indicated genes, Pearson correlation coefficient r=-0.05, p=0.56 (two-sided).

Supplemental Tab. 1: Clinical and pathological features of patients enrolled in the plasmaMATCH trial with ctDNA sequencing data

	N=800	
	n	%
Age group (years) at registration		
<50	215	26.9
50-59	263	32.9
60-69	209	26.1
≥70	113	14.1
Metastatic disease present at diagnosis	117	14.6
Time since primary diagnosis (years)		
<1 year	35	4.4
1-3 years	170	21.2
3-5 years	153	19.1
≥5 years	421	52.6
Not known/Missing	21	2.6
Tumor characteristics at initial diagnosis:		
Pathological invasive tumor size (cm)		
≤2cm	223	29.1
2-5cm	252	31.5
>5cm	87	10.9
Not known/Missing	238	29.8
Nodal status		
N0	212	26.5
N1-3	191	23.9
N4+	164	20.5
Not known/Missing	233	29.1
Histological type		
Ductal	577	72.1
Lobular	79	9.9
Mixed ductal & lobular	30	3.8
Other invasive	15	1.9
DCIS	2	0.3
Not known/Missing	97	12.1
Tumor grade		
G1	35	4.4
G2	319	39.9

	N=800	
	n	%
G3	322	40.3
Not known/Missing	122	15.3
Molecular subtype		
HR+, HER2-	515	64.4
HR+, HER2+	46	5.8
HR-, HER2+	26	3.3
TNBC	138	17.3
HR+, HER2 unknown	31	3.9
Other	9	1.1
Not known/Missing	35	4.4
Disease sites		
Visceral	627	78.4
Soft tissue/nodal	143	17.8
Bone Only	11	1.4
Treatment received for locally advanced/metastatic disease prior to study registration		
Chemotherapy	542	67.8
1 line	*283*	35.4
2 lines	*152*	19.0
>2 lines	*107*	13.4
Endocrine therapy	520	65.0
1 line	*253*	31.6
2 lines	*168*	21.0
3 lines	*91*	11.4
>3 lines	*8*	1.0
Total lines of treatment received (chemotherapy and endocrine therapy combined)		
0	77	9.6
1	220	27.5
2	189	23.6
3	134	16.8
4	84	10.5
5	57	7.1
>5	39	4.9
Other systemic therapy		
Anti-HER2 therapy	*64*	8.0
mTOR inhibitor (everolimus, vistusertib)	*93*	11.6

	N=800	
	n	*%*
CDK4/6 inhibitor (palbociclib, ribociclib, abemaciclib)	*77*	9.6
Immunotherapy (atezolizumab, pembrolizumab)	*15*	1.9
Denosumab	*68*	8.5
Bisphosphonate	*38*	4.8
Other	*33*	4.1

Supplementary Tab. 2: Comparison of MSK-IMPACT and plasmaMATCH cohorts

Breast Cancer Subtype		plasmaMATCH			MSK-IMPACT			p value
		n cases	n	%	n cases	n	%	
HR+HER2-	Ductal	376	515	73.0	428	584	73.3	0.0003
	Lobular	63	515	12.2	107	584	18.3	
	Other/missing	76	515	14.8	49	584	8.4	
HR+HER2+	Ductal	35	46	76.1	56	75	74.7	0.28
	Lobular	2	46	4.3	9	75	12.0	
	Other/missing	9	46	19.6	10	75	13.3	
HR-HER2+	Ductal	20	26	76.9	35	43	81.4	0.62
	Lobular	1	26	3.8	3	43	7.0	
	Other/missing	5	26	19.2	5	43	11.6	
TNBC	Ductal	110	138	79.7	125	151	82.8	0.04
	Lobular	6	138	4.3	14	151	9.3	
	Other/missing	22	138	15.9	12	151	7.9	

p values from Chi-squared test

Supplementary Tab. 3: Comparison of Clinico-pathological characteristics of patients with and without ctDNA alterations.

Clinical Characteristic		Patients with alterations		Patients without alterations		p value
		n = 743		n = 57		
		n	%	n	%	
Breast cancer subtype	HR+HER2-	484	65.1	31	54.4	0.10
	HR+HER2+	40	5.4	6	10.5	
	HR-HER2+	22	3.0	4	7.0	
	TNBC	130	17.5	8	14.0	
	Unknown	67	9.0	8	14.0	
Histology	Ductal	534	71.9	43	75.4	0.83
	Lobular	75	10.1	4	7.0	
	Other	43	5.8	4	7.0	
	Not known	91	12.2	6	10.5	
Disease burden	Visceral	586	78.9	41	71.9	0.41
	Soft tissue/nodal	131	17.6	12	21.1	
	Bone	10	1.3	1	1.8	
	Not known	16	2.2	3	5.3	
Number of lines prior treatment	0	67	9.0	10	17.5	0.02
	1-2	374	50.3	35	61.4	
	3-4	209	28.1	9	15.8	
	5+	93	12.5	3	5.3	

p values from Chi-squared test

Supplementary Tab. 4. Primers used for ddPCR assessment of mutation status for plasmaMATCH screening and sequencing validation

Assay use	Gene	Mutation	Alteration	Singleplex/multiplex	Assay ID	Primer F	Sequence	Primer R	Sequence	WT Probe	Mutant Probe
PlasmaMATCH trial screening	*PIK3CA*	E542K	c.1624G>A	Multiplex		PIK3CA.E542K_fw	AAGCAATTTCTACACGAGA	PIK3CA.E542K_rev	GTGCACTTACCTGTGAC	TCTCTGAAATCACTGAGCAG	TCTCTCTAAAATCACTGAGCA
		E545K	c.1633G>A	Multiplex		PIK3CA.E545K_fw	AAGCAATTTCTACACGAGA	PIK3CA.E545K_rev	GTGCACTTACCTGTGAC	TCTCTGAAATCACTGAGCAG	CTGAAATCACTAAGCAGGAG
		H1047R	c.3140A>G	Multiplex		PIK3CA.H1047R_fw	AAGAGGCTTTGGAGTATTTC	PIK3CA.H1047R _rev	CCAATCCATTTTTGTTGTCC	TGCACATCATGGTGGC	ATGCACGTCATGGTGG
		H1047L	c.3140A>T	Multiplex		PIK3CA.H1047L_fw	AAGAGGCTTTGGAGTATTTC	PIK3CA.H1047L _rev	CCAATCCATTTTTGTTGTCC	TGCACATCATGGTGGC	ATGCACGTCATGGTGG
	ESR1	E380Q	c.1138G>C	Multiplex	dHsaMDXE91450042						
		L536R	c.1607T>G	Multiplex	dHsaMDXE91450042						
		Y537C	c.1610A>G	Multiplex	dHsaMDXE91450042						

		D538G	c.1613A>G	Multiplex	dHsaMDXE91450042						
	ESR1	S463P	c.1387T>C	Multiplex	dHsaMDXE65719815						
		Y537N	c.1609T>A	Multiplex	dHsaMDXE65719815						
		Y537S	c.1610A>C	Multiplex	dHsaMDXE65719815						
	AKT1	E17K	c.49G>A	Singleplex	dHsaCP2000031 and dHsaCP2000032						
	HER2	S310F	c.929C>T	Singleplex		ERBB2.S310F_fw	CTCCTTAGACAACTACCTTTC	ERBB2.S310F_rev	GTTGTGCAGGGGGC	AGGGTGCAGGATCCC	AGGGTGCAGAATCCCA

	HER2	S310Y	c.929C>A	Singleplex		ERBB2.S310Y_fw	CTCCTTAGACAACTACCTTTC	ERBB2.S310Y_rev	GTTGTGCAGGGGGC	AGGGTGCAGGATCCC	AGGGTGCAGTATCCCA
	HER2	L755S	c.2264T>C	Singleplex		ERBB2.755_fw	GAGAATGTGAAAATTCCAGTG	ERBB2.755_rev	TAGCAGGAGAGGGTGG	TGTTTTCCCTCAACACTTTG	TCCCTCGACACTTTGATG
	HER2	V777L	c.2329G>T	Singleplex		ERBB2.777_fw	CAGCGTACCCTTGTCC	ERBB2.777_rev	AGAAGGCGGGAGACAT	TGGCTGGTGTGGGC	ATGGCTGGTTTGGGC
	HER2	P780_Y781insGSP	c.2339_2340ins	Singleplex		ERBB2.GSP_fw	CAGCGTACCCTTGTCCC	ERBB2.GSP_rev	GTCAGGCAGATGCCCAGA	TGCTTCGTGCACACGGTGC	GGGCTCCCCGGGCTCCC
	HER2	A775_G776insYVMA	c.2325_2326ins	Singleplex		ERBB2.YVMA_fw	CAGCGTACCCTTGTCCC	ERBB2.YVMA_rev	GTCAGGCAGATGCCCAGA	TGCTTCGTGCACACGGTGC	ATACGTGATGGCTTACGTGATGGCTG
Mutation Validation	*PIK3CA*	E545K	c.1633G>A	Singleplex		PIK3CA.E545K_fw	AAGCAATTTCTACACGAGA	PIK3CA.E545K_rev	GTGCACTTACCTGTGAC	TCTCTGAAATCACTGAGCAG	CTGAAATCACTAAGCAGGAG
	PIK3CA	E542Q	c.1624G>C	Singleplex		PMV_01_F	AGCTCAAAGCAATTTCTACACGAGAT	PMV_01_R	CTGTGACTCCATAGAAAATCTTTCTCCT	CCTCTCTCTGAAATCA	CCTCTCTCTAAAATCA
	PIK3CA	E726K	c.2176G>A	Singleplex		PMV_03_F	CATTAACTTAACTGACATTCTCAAACAGG	PMV_03_R	AACACAAACTAGAGTCACACACCTTT	AGAAGAAGGATGAAACAC	AGAAGAAGGATAAAACAC
	PIK3CA	E545Q	c.1633G>C	Singleplex		PMV_02_F	TCAAAGCAATTTCTACACGAGATCCT	PMV_02_R	CACTTACCTGTGACTCCATAGAAAATCT	TCTCCTGCTCAGTGATT	CTCCTGCTGAGTGATT
	PIK3CA	H1047R	c.3140A>G	Singleplex		PIK3CA.H1047R_fw	AAGAGGCTTTGGAGTATTTC	PIK3CA.H1047R _rev	CCAATCCATTTTTGTTGTCC	TGCACATCATGGTGGC	ATGCACGTCATGGTGG

	HER2	G727A	c.2180G>C	Singleplex		PMV_07_F	AGACGGAGCTGAGGAAGGT	PMV_07_R	CCTTGTAGACTGTGCCAAAAGC	AAGGTGCTTGGATCTG	AAGGTGCTTGCATCTG
	HER2	E717D	c.2151G>C	Singleplex		PMV_06_F	GCGCAGATGCGGATCCT	PMV_06_R	CAGATCCAAGCACCTTCACCTT	TCAGCTCCGTCTCTTT	AGCTCCGTGTCTTT
	HER2	Q711H	c.2133G>C	Singleplex		PMV_05_F	GGAGCGATGCCCAACCA	PMV_05_R	CCTCAGCTCCGTCTCTTTCAG	ATCCGCATCTGCGCC	CCGCATGTGCGCC
	HER2	L786V	c.2356C>G	Singleplex		PMV_09_F	TGTGGGCTCCCCATATGTCT	PMV_09_R	GCTGCACCGTGGATGTCA	CCGCCTTCTGGGCAT	CGCCTTGTGGGCAT
	HER2	I628M	c.1884C>G	Singleplex		PMV_04_F	GGAGGGCGCATGCCA	PMV_04_R	GCAGAAAAGACCGTTGGACTCA	TTGCCCCATCAACTG	TTGCCCCATGAACTG
	HER2	D1105N	c.3313G>A	Singleplex		PMV_14_F	GGGCTGCAAAGCCTC	PMV_14_R	TCCTCACTGTACCGCTGTAGA	CCCACACATGACCCCAG	CCCACACATAACCCCAG
	HER2	S1002N	c.3005G>A	Singleplex		PMV_12_F	GGGCCCAGCCAGTCC	PMV_12_R	CATCGTCCTCCAGCAGTGA	TTGGACAGCACCTTC	CTTGGACAACACCTTC
	HER2	L800R	c.2399T>G	Singleplex		PMV_10_F	GGCATCTGCCTGACATCCA	PMV_10_R	GTTTTCCCGGACATGGTCTAAGAG	ACACAGCTTATGCCC	ACAGCGTATGCCC
	HER2	L800P	c.2399T>C	Singleplex		PMV_11_F	GGCATCTGCCTGACATCCA	PMV_11_R	GTTTTCCCGGACATGGTCTAAGAG	TGACACAGCTTATGCC	TGACACAGCCTATGCC

	HER2	Q1206K	c.3616C>A	Singleplex		PMV_16_F	GGAGAACCCCGAGTACTTGAC	PMV_16_R	GGCTGAAGGCAGGAGGAG	TGCCCCTCAGCCCCA	TGCCCCTAAGCCCCA
	HER2	R1153Q	c.3458G>A	Singleplex		PMV_15_F	CAGAATATGTGAACCAGCCAGATGT	PMV_15_R	GGGCAGCAGGCAGAGG	CCTTCGCCCCGAGAGG	CTTCGCCCCAAGAGG
	HER2	E1079K	c.3235G>A	Singleplex		PMV_13_F	AGGCCCCCAGGTCTCC	PMV_13_R	TTCCCAGGTCACCATCAAATACATC	CTGGCACCCTCCGAAGG	TGGCACCCTCCAAAGG
	HER2	V777M	c.2329G>A	Singleplex		PMV_08_F	GTCCCCAGGAAGCATACGT	PMV_08_R	CAGAAGGCGGGAGACATATGG	ATGGCTGGTGTGGGCT	ATGGCTGGTATGGGCT
	HER2	D769H	c.2305G>C	Singleplex		2305G_C_F	ACATCCCCCAAAGCCAACAAA	2305G_C_R	GTCCTTCCTGTCCTCCTAGCA	CTTACGTCTAAGATTTC	TTACGTGTAAGATTTC
	HER2	D769Y	c.2305G>T	Singleplex		2305G_T_F	GGAAAACACATCCCCCAAAGC	2305G_T_R	GTCCTTCCTGTCCTCCTAGCA	CAAAGAAATCTTAGACGTAAGC	CAAAGAAATCTTATACGTAAGC
	HER2	L755S	c.2264T>C	Singleplex		ERBB2.755_fw	GAGAATGTGAAAATTCCAGTG	ERBB2.755_rev	TAGCAGGAGAGGGTGG	TGTTTTCCCTCAACACTTTG	TCCCTCGACACTTTGATG
	HER2	I767M	c.2301C>G	Singleplex		ERBB2.767_fw	GAGAATGTGAAAATTCCAGTG	ERBB2.767_rev	TAGCAGGAGAGGGTGG	AAGAAATCTTAGACGTAAGCC	AAGAAATGTTAGACGTAAGCC
	HER2	S310F	c.929C>T	Singleplex		ERBB2.S310F_fw	CTCCTTAGACAACTACCTTTC	ERBB2.S310F_rev	GTTGTGCAGGGGGC	AGGGTGCAGGATCCC	AGGGTGCAGAATCCCA

Supplementary References

1. Turner, N.C., *et al.* Circulating tumour DNA analysis to direct therapy in advanced breast cancer (plasmaMATCH): a multicentre, multicohort, phase 2a, platform trial. *The Lancet Oncology*.
2. Lanman, R.B., *et al.* Analytical and Clinical Validation of a Digital Sequencing Panel for Quantitative, Highly Accurate Evaluation of Cell-Free Circulating Tumor DNA. *PLoS One* **10**, e0140712 (2015).
3. Comprehensive molecular portraits of human breast tumours. *Nature* **490**, 61-70 (2012).
4. Gulhan, D.C., Lee, J.J.-K., Melloni, G.E.M., Cortés-Ciriano, I. & Park, P.J. Detecting the mutational signature of homologous recombination deficiency in clinical samples. *Nature Genetics* **51**, 912-919 (2019).
5. Zhou, X., *et al.* Exploring genomic alteration in pediatric cancer using ProteinPaint. *Nature Genetics* **48**, 4-6 (2016).
