# Supplementary material for: Genomic profile of advanced breast cancer in circulating tumour DNA
Source: Nat Commun. 2021 Apr 23;12:2423. doi: 10.1038/s41467-021-22605-2 (PMC8065112; doi:10.1038/s41467-021-22605-2)
Supplement: Supplementary file 3 — Description of Additional Supplementary Files [file 41467_2021_22605_MOESM3_ESM.pdf]

Description of Additional Supplementary File

File name: Supplementary Data 1

Description: Targeted sequencing data of 800 patients enrolled in plasmaMATCH

Sheet 1: Summary matrix. Mutation positive (‘1’) or negative (‘0’) for all patients (rows) and genes (columns). Includes pathogenic SNVs and indels.

Sheet 2: SNVs and indels. Includes the judgement of whether the mutation call was considered pathogenic (‘pathogenic’, see “Methods”).

Sheet 3: CNVs.

Sheet 4: Fusions.
