# Supplementary material for: Genomic profile of advanced breast cancer in circulating tumour DNA
Source: Nat Commun. 2021 Apr 23;12:2423. doi: 10.1038/s41467-021-22605-2 (PMC8065112; doi:10.1038/s41467-021-22605-2)
Supplement: Supplementary file 5 — Reporting Summary [file 41467_2021_22605_MOESM5_ESM.pdf]

nature research

Corresponding author(s): Nicholas Turner
Last updated by author(s): 16/02/21

Reporting Summary

Nature Research wishes to improve the reproducibility of the work that we publish. This form provides structure for consistency and transparency in reporting. For further information on Nature Research policies, see our Editorial Policies and the Editorial Policy Checklist.

Statistics

For all statistical analyses, confirm that the following items are present in the figure legend, table legend, main text, or Methods section.

n/a	Confirmed	
☐	☒	The exact sample size (n) for each experimental group/condition, given as a discrete number and unit of measurement
☒	☐	A statement on whether measurements were taken from distinct samples or whether the same sample was measured repeatedly
☐	☒	The statistical test(s) used AND whether they are one- or two-sided<br>*Only common tests should be described solely by name; describe more complex techniques in the Methods section.*
☒	☐	A description of all covariates tested
☐	☒	A description of any assumptions or corrections, such as tests of normality and adjustment for multiple comparisons
☐	☒	A full description of the statistical parameters including central tendency (e.g. means) or other basic estimates (e.g. regression coefficient) AND variation (e.g. standard deviation) or associated estimates of uncertainty (e.g. confidence intervals)
☐	☒	For null hypothesis testing, the test statistic (e.g. F, t, r) with confidence intervals, effect sizes, degrees of freedom and P value noted<br>*Give P values as exact values whenever suitable.*
☒	☐	For Bayesian analysis, information on the choice of priors and Markov chain Monte Carlo settings
☒	☐	For hierarchical and complex designs, identification of the appropriate level for tests and full reporting of outcomes
☐	☒	Estimates of effect sizes (e.g. Cohen's d, Pearson's r), indicating how they were calculated

Our web collection on statistics for biologists contains articles on many of the points above.

Software and code

Policy information about availability of computer code

Data collection	Microsoft excel 2016 was used to collect data

Data analysis

All analyses were undertaken with reference to genome reference hg19.
The following software was used within a bioinformatic pipeline to annotate the sequencing data provided by Guardant Health:
vcf2maf (https://github.com/mskcc/vcf2maf)
VEP (version 96, https://www.ensembl.org/info/docs/tools/vep/index.html)
OncoKB (version 1, https://github.com/oncokb/oncokb-annotator)

Data was filtered with reference to the following databases
Genome Aggregation Database (https://gnomad.broadinstitute.org/)
CancerHotspots (version 2, https://www.cancerhotspots.org/#/download)
Cosmic (version 90, https://cancer.sanger.ac.uk/cosmic)

Data was analysed using R (version 3.5.2), and Graphpad Prism (version 8.0.1).
Pairwise Fishers Exact tests were undertaken using the the fishers.multicomp() function from R package RVAideMemoire (version 0.9.74).
Pairwise Kruskal-Wallis tests were undertaken using the pairw.kw() function from R package asbio (version 1.5.5).
deconstructSigs (version 1.8.0) and SigMA (downloaded 30th September 2020) were used to analyse mutational signatures.
Plots were created using Graphpad Prism (version 8.0.1) and the R software packages ggplot2 (version 3.2.1), pheatmap (version 1.0.12), ggalluvial (version 0.11.1) and survminer (version 0.4.6).

For manuscripts utilizing custom algorithms or software that are central to the research but not yet described in published literature, software must be made available to editors and reviewers. We strongly encourage code deposition in a community repository (e.g. GitHub). See the Nature Research guidelines for submitting code & software for further information.

Data

Policy information about availability of data

All manuscripts must include a data availability statement. This statement should provide the following information, where applicable:

- Accession codes, unique identifiers, or web links for publicly available datasets
- A list of figures that have associated raw data
- A description of any restrictions on data availability

The plasmaMATCH Guardant360 sequencing data generated and analysed during the current study are available within Supplementary Data 1. To protect the privacy and confidentiality of patients in this study, clinical data are not made publicly available in a repository or the supplementary material of the article. Any requests for further data will be subject to approval of a formal data access request in accordance with the Institute of Cancer Research Clinical Trials and Statistics Unit (ICR-CTSU) data and sample access policy. Trial documentation including the protocol are available on request by contacting plasmamatch-icrctsu@icr.ac.uk. The ICR-CTSU supports the wider dissemination of information from the research it conducts, and increased cooperation between investigators. Trial data are collected, managed, stored, shared, and archived according to ICR-CTSU standard operating procedures to ensure the enduring quality, integrity, and utility of the data. Formal requests for data sharing are considered in line with ICR-CTSU procedures, with due regard given to funder and sponsor guidelines. Requests are to be made via a standard proforma describing the nature of the proposed research and extent of data requirements. Data recipients are required to enter a formal data sharing agreement, which describes the conditions for data release and requirements for data transfer, storage, archiving, publication, and intellectual property. Requests are reviewed by the trial management group in terms of scientific merit and ethical considerations including patient consent. Data sharing is undertaken if proposed projects have a sound scientific or patient benefit rationale, as agreed by the trial management group and approved by the trial steering committee, as required. Restrictions relating to patient confidentiality and consent will be lessened by aggregating and anonymising identifiable patient data. Additionally, all indirect identifiers that may lead to deductive disclosures will be removed in line with Cancer Research UK data sharing guidelines.

The MSKCC data are available in the cBioPortal for Cancer Genomics database (https://www.cbioportal.org/study/summary?id=breast_msk_2018).

The TCGA data are available in the cBioPortal for Cancer Genomics database (https://www.cbioportal.org/study/summary?id=brca_tcga_pan_can_atlas_2018). TGCA data analysed for this manuscript were released 28th January 2016.

Field-specific reporting

Please select the one below that is the best fit for your research. If you are not sure, read the appropriate sections before making your selection.

☒ Life sciences ☐ Behavioural & social sciences ☐ Ecological, evolutionary & environmental sciences

For a reference copy of the document with all sections, see nature.com/documents/nr-reporting-summary-flat.pdf

Life sciences study design

All studies must disclose on these points even when the disclosure is negative.

Sample size

Sequencing data was obtained from 800 patients within the plasmaMATCH trial. This is the number of patients for whom we had available sequencing data at the time of data cutoff.

Data exclusions

No data was excluded from the analysis

Replication

The mutational signature analysis using deconstructSigs was bootstrap sampled with 200 iterations for each subtype to generate confidence

	intervals. Replication is not necessary for sequencing data generated by Guardant Health for plasmaMATCH. Extensive validation was undertaken using the orthogonal approach of ddPCR, as described in the manuscript.
Randomization	plasmaMATCH is a phase IIa non-randomised open-label trial. This ad-hoc analysis concerns the sequencing data generated from all women screened for trial entry, and as such is not randomised as all patients with available sequencing data were included.
Blinding	The plasmaMATCH trial was not blinded. Breast cancer phenotype information was available to the investigators during the genomic analysis and no patients were excluded, so there was no requirement to blind the analysis.

Reporting for specific materials, systems and methods

We require information from authors about some types of materials, experimental systems and methods used in many studies. Here, indicate whether each material, system or method listed is relevant to your study. If you are not sure if a list item applies to your research, read the appropriate section before selecting a response.

Materials & experimental systems

n/a	Involved in the study
☒	☐ Antibodies
☒	☐ Eukaryotic cell lines
☒	☐ Palaeontology and archaeology
☒	☐ Animals and other organisms
☐	☒ Human research participants
☐	☒ Clinical data
☒	☐ Dual use research of concern

Methods

n/a	Involved in the study
☒	☐ ChIP-seq
☒	☐ Flow cytometry
☒	☐ MRI-based neuroimaging

Human research participants

Policy information about studies involving human research participants

Population characteristics	This manuscript presents an analysis of the genomic sequencing results of patients recruited within the plasmaMATCH trial. The plasmaMATCH trial clinical results have been published previously. The trial recruited patients with advanced breast cancer (ABC) with measurable disease, who had progressed on prior therapy for ABC, or relapsed within 12 months of adjuvant chemotherapy, and (following an amendment partway through the trial) had not had more than two lines of chemotherapy for ABC. Of 1051 patients recruited to the trial, 800 patients had available sequencing data and are presented in the manuscript. Of these patients, 64.4% (N=515) had hormone receptor positive (HR+) HER2 negative (HER2-, lack of HER2 over-expression and/or gene amplification) disease, 9.1% (N=72) were HER2 positive, and 17.3% (N=138) had triple-negative breast cancer (TNBC). 67.8% of patients had recieved chemotherapy in the metastatic setting, with 13.4% having received more than 2 lines of prior chemotherapy. A full table of baseline characteristics of the 800 patients is provided in the manuscript.
Recruitment	Patients were recruited to take part in the trial from 18 recruitment centers across the UK. Patients entered a treatment cohort following ctDNA testing if they possessed a mutation with an available matched targeted therapy . Therefore physicians may have more heavily recruited patients in whom they have prior knowledge that they harbour targetable mutations making them eligible for targeted therapy within the plasmaMATCH trial. As DNA sequencing is not widely available in the NHS, this is unlikely to have significantly biased the recruitment to the trial or altered the results presented in the manuscript.
Ethics oversight	South Central - Oxford C Research Ethics Committee, 20/07/2016, ref: 16/SC/0271

Note that full information on the approval of the study protocol must also be provided in the manuscript.

Clinical data

Policy information about clinical studies

All manuscripts should comply with the ICMJEguidelines for publication of clinical research and a completedCONSORT checklist must be included with all submissions.

Clinical trial registration	NCT03182634
Study protocol	The full trial protocol is available within the supplementary appendix of the clinical trial publication, accessed here: https://www.thelancet.com/journals/lanonc/article/PIIS1470-2045(20)30444-7/fulltext
Data collection	Patients were recruited to take part in the trial from 18 recruitment centers (hospitals) across the UK. Between Dec 21, 2016, and April 26, 2019, 1051 patients were registered into the study, with targeted sequencing results were available for 800 patients.
Outcomes	The primary and secondary endpoints of the plasmaMATCH trial were pre-defined in a statistical analysis plan. The primary endpoint of the trial was confirmed objective response rate in cohorts A-D. The trial has published its clinical findings, and this can be accessed here: www.thelancet.com/journals/lanonc/article/PIIS1470-2045(20)30444-7/fulltext.
